# Novel Insights into the Cellular Localization and Regulation of the Autophagosomal Proteins LC3A, LC3B and LC3C

**DOI:** 10.3390/cells9102315

**Published:** 2020-10-18

**Authors:** Marius W. Baeken, Katja Weckmann, Philip Diefenthäler, Jan Schulte, Kamran Yusifli, Bernd Moosmann, Christian Behl, Parvana Hajieva

**Affiliations:** 1 Institute of Pathobiochemistry, University Medical Center of the Johannes Gutenberg University, 55131 Mainz, Germany; mbaeken@uni-mainz.de (M.W.B.); katja_weckmann@yahoo.de (K.W.); philip.diefenthaeler@gmail.com (P.D.); jan.schulte@artorg.unibe.ch (J.S.); yusiflik@gmail.com (K.Y.); moosmann@uni-mainz.de (B.M.); cbehl@uni-mainz.de (C.B.); 2 Institute for Molecular Medicine, MSH Medical School, 20457 Hamburg, Germany

**Keywords:** ATG8, autophagy, GABARAP, LC3A, LC3B, LC3C, sequestosome 1 (p62), sirtuin 1

## Abstract

Macroautophagy is a conserved degradative process for maintaining cellular homeostasis and plays a key role in aging and various human disorders. The microtubule-associated protein 1A/1B light chain 3B (MAP1LC3B or LC3B) is commonly analyzed as a key marker for autophagosomes and as a proxy for autophagic flux. Three paralogues of the LC3 gene exist in humans: LC3A, LC3B and LC3C. The molecular function, regulation and cellular localization of LC3A and LC3C have not been investigated frequently, even if a similar function to that described for LC3B appears likely. Here, we have selectively decapacitated LC3B by three separate strategies in primary human fibroblasts and analyzed the evoked effects on LC3A, LC3B and LC3C in terms of their cellular distribution and co-localization with p62, a ubiquitin and autophagy receptor. First, treatment with pharmacological sirtuin 1 (SIRT1) inhibitors to prevent the translocation of LC3B from the nucleus into the cytosol induced an increase in cytosolic LC3C, a heightened co-localization of LC3C with p62, and an increase LC3C-dependent autophagic flux as assessed by protein lipidation. Cytosolic LC3A, however, was moderately reduced, but also more co-localized with p62. Second, siRNA-based knock-down of SIRT1 broadly reproduced these findings and increased the co-localization of LC3A and particularly LC3C with p62 in presumed autophagosomes. These effects resembled the effects of pharmacological sirtuin inhibition under normal and starvation conditions. Third, siRNA-based knock-down of total LC3B in cytosol and nucleus also induced a redistribution of LC3C as if to replace LC3B in the nucleus, but only moderately affected LC3A. Total protein expression of LC3A, LC3B, LC3C, GABARAP and GABARAP-L1 following LC3B decapacitation was unaltered. Our data indicate that nuclear trapping and other causes of LC3B functional loss in the cytosol are buffered by LC3A and actively compensated by LC3C, but not by GABARAPs. The biological relevance of the potential functional compensation of LC3B decapacitation by LC3C and LC3A warrants further study.

## 1. Introduction

Macroautophagy is an important catabolic process for maintaining cellular homeostasis. Misfolded, old, or damaged proteins and organelles are captured in autophagosomes—vesicles with a double membrane—and are subsequently degraded by fusion with lysosomes [1,2]. The degraded products like amino acids or fatty acids can further be metabolized to generate energy, or they can be recycled to protein or membrane biogenesis. Autophagy is particularly important and upregulated during metabolic stress, including glucose reduction and other bioenergetic deficiencies [3]. Impaired autophagy plays a major role in aging and various human disorders, including many neurodegenerative disorders [4,5]. 

Autophagy is a highly regulated process involving the controlled expression of autophagy-related genes (atg). The formation of autophagosomes starts with the generation of a double lipid membrane, which subsequently matures from an early phagophore into a mature autophagosome containing lipidated ATG microtubule-associated proteins 1A/1B light chain 3 proteins (MAP1LC3 proteins, LC3 proteins). Three members of the LC3 family occur in humans: LC3A, LC3B, and LC3C, of which LC3B is widely used as a marker for autophagy and a tool to assess general autophagic flux [6]. LC3A and LC3C are suggested to have similar functions to LC3B, but might also be involved in autophagy-unrelated phenomena such as signal transduction or viral replication [7,8,9]. Their precise molecular mode of action remains unknown. 

The members of the LC3 protein family are generated from different mRNA transcripts that are derived from distinct genomic locations, but they share high sequence similarity [9]. LC3B is localized to the nucleus as well as to the cytoplasm and has been shown to cycle between these cellular compartments. This process is regulated by lysine acetylation and deacetylation dependent on the class III histone deacetylase (HDAC), sirtuin 1 (SIRT1) [10]. Moreover, LC3B is present in a non-lipidated (LC3B-I) and a lipidated (LC3B-II) form. In the cytoplasm, deacetylated LC3B-I is lipidated by conjugation with phosphatidylethanolamine and thereby converted to LC3B-II, which in turn is recruited by autophagosomes. Whether LC3A and LC3C are regulated in a similar manner is still unknown. Besides the LC3s, three further members of the ATG8 protein family exist in humans: GABARAP, GABARAP-L1, and GABARAP-L2, which are thought to be mainly involved in late steps of autophagy, such as autophagosome elongation and autophagosome-to-lysosome fusion [11,12].

To gain further insight into the cellular roles of the different LC3s under near-physiological conditions, we analyzed their distribution (nuclear versus cytosolic) and their degree of co-localization with sequestosome1 (p62), a major ubiquitin and autophagy receptor, in response to an experimentally induced decapacitation of LC3B, the canonical and best-characterized LC3. LC3B decapacitation was achieved (i) with a selective SIRT1 inhibitor (Ex527) under normal and starvation conditions; trichostatin A (TSA), an inhibitor of non-sirtuin HDACs, was studied as an alternative pro-acetylation agent. Beyond these pharmacological treatments, we used (ii) siRNA-mediated knock-down experiments to reduce the total expression of SIRT1. Finally, (iii) the effects of siRNA-mediated knock-down of all three LC3 paralogues were investigated. Primary human diploid fibroblasts were chosen for these investigations since they offer the study of physiological regulatory events in a genetically undisturbed, human setting of unreduced complexity.

In all cases, we subsequently studied the cellular localization and expression of LC3A, LC3B and LC3C under Bafilomycin A1 (BafA1) treatment. BafA1 is an inhibitor of V-ATPase that prevents autophagosome-lysosome fusion and autophagolysosome acidification. The observed differential behavior of the ATG8 protein family implies a substantial, although incomplete mutual functional compensation of the three LC3 family members. Comparison of conserved regions, binding sites, and recognized LIR motifs supported the idea that a sub- or neo-functionalization of the LC3 protein family has occurred during evolution.

## 2. Materials and Methods

### 2.1. Cell Culture

Cell culture media and supplements, including high-glucose Dulbecco’s Modified Eagle’s Medium (DMEM), Phosphate-Buffered Saline (PBS) and Fetal Calf Serum (FCS) were obtained from Invitrogen (Thermo Fisher Scientific, Waltham, MA, USA). Laboratory chemicals including Ex527 (6-chloro-2,3,4,9-tetrahydro-1H-carbazole-1-carboxamide) and trichostatin A were purchased from Sigma (Sigma-Aldrich, St. Louis, MO, USA) at the highest purity available. Bafilomycin A1 (BafA1) was from Biozol (Eching, Germany).

Primary human fibroblasts (IMR90 cells) were obtained from the Coriell Institute for Medical Research (Camden, NJ, USA) and cultivated as described previously [13]. In brief, the cells were maintained in phenol red-containing DMEM with high glucose (4.5 g/L) supplemented with 10% heat-inactivated FCS, 1 mM sodium pyruvate, and 1x antibiotic/antimycotic mixture (order number LS-B5909, LSBio, Eching, Germany). Starvation experiments were performed by replacing the standard medium with Earle’s balanced salt solution (Sigma-Aldrich, St. Louis, MO, USA; order number E2888) for 4 h preceding the analysis. For routine culture, cells were grown on 10 cm dishes, maintained at 37 °C in a humidified atmosphere containing 5% CO_2_, and were passaged twice a week. Experiments were carried out with cells at population doublings (PDLs) between 20 and 33. For Western blotting experiments, cells were plated at a density of 0.4 × 10^6^ cells per 10 cm dish and grown in full medium for 24 h. The cells were subsequently washed with pre-warmed PBS and new medium was added (10 mL), followed by treatment for 48 h with 10 nM TSA (in DMSO), 100 nM Ex527 (in DMSO), or 10 µL DMSO as vehicle control. Four hours prior to harvest, 2 µM BafA1 was added where appropriate. All treatment groups were visually inspected using light microscopy by two independent researchers to avoid the analysis of morphologically damaged cells.

### 2.2. Western Blotting

Treated cells were washed with ice-cold PBS and harvested, with brief sonication, in lysis buffer (50 mM Tris-HCl, pH 6.8; 2% sodium dodecyl sulfate (SDS); 10% sucrose; and EDTA-free protease inhibitor cocktail (order number 5056489001 from Roche, Basel, Switzerland)). Protein concentration was determined using a BCA kit (Thermo Fisher Scientific, Waltham, MA, USA) following the manufacturer’s protocol. An amount of 10 μg protein was loaded on a 12–15% SDS gel and separated by electrophoresis. Afterwards, the proteins were transferred onto a nitrocellulose membrane and blocked with 4% non-fat milk in PBST. Individual proteins were detected with the following primary antibodies: rabbit anti-LC3A (1:1000, from Cell Signaling, Danvers, MA, USA; order number 4599), rabbit anti-LC3B (1:1000, from Sigma-Aldrich, St. Louis, MO, USA; order number L7543), rabbit anti-LC3C (1:1000, from Abcam, Cambridge, UK; order number ab150367), mouse anti-GABARAP (1:1000, from MBL, Woburn, MA, USA; order number M135-3), rabbit anti-GABARAP-L1 (1:1000, from Cell Signaling, Danvers, MA, USA; order number 26632), rabbit anti-GABARAP-L2 (1:1000, from Cell Signaling, Danvers, MA, USA; order number 14256), mouse anti-SIRT1 (1:1000, from Cell Signaling, Danvers, MA, USA; order number 8469), guinea pig anti-p62 (1:1000, from Progen, Heidelberg, Germany; order number GP62-C), mouse anti-α-tubulin (1:1000, from Sigma-Aldrich, St. Louis, MO, USA; order number T9026). The immunoreactive signal of α-tubulin was used as control for equal protein loading. Primary antibodies were detected with horseradish peroxidase-conjugated secondary antibodies (1:5000, from Jackson Immunoresearch, West Grove, PA, USA). Immunoreactive bands were developed using commercial kits (Enhanced Chemiluminescence Plus from Amersham Pharmacia Biotech, Piscataway, NJ, USA) and scanned with an Amersham Imager 600. The densitometric analyses of the developed bands were done with Aida Image Analysis Software (Raytest, Straubenhardt, Germany).

### 2.3. Antibody Validation

Recombinant His-tagged LC3A, untagged LC3B and GST-tagged LC3C were purchased from Novus Biologicals (Centennial, CO, USA). The recombinant human LC3A protein (order number NBP1-45308), fused to a His-tag at the C-terminus, and the recombinant human LC3B protein (order number NBP2-50960) was expressed in *E. coli*. The recombinant human LC3C protein (order number H00440738-P01) with a GST-tag at the N-terminus had been expressed in wheat germ. The LC3 proteins were diluted in Western blotting lysis buffer to contain a total amount of 10 ng, 1 ng or 0.1 ng protein together with 20 µg milk proteins. Samples were supplemented with loading buffer and loaded several times onto a gel without boiling. Western blotting was performed in the same manner as described in 2.2. After blotting, the membranes were cut vertically to allow parallel incubation with antibodies against LC3A, LC3B, and LC3C using rabbit anti-LC3A (1:1000, from Cell Signaling, Danvers, MA, USA; order number 4599), rabbit anti-LC3B (1:1000, Sigma-Aldrich, St. Louis, MO, USA; order number L7543), rabbit anti-LC3C (1:1000, from Abcam, Cambridge, UK; order number ab150367). The membrane strips carrying identical LC3s were then developed in parallel using enhanced chemiluminescence as before.

### 2.4. Immunoprecipitation

Cells cultivated in 15 cm dishes were treated as appropriate, washed with ice-cold PBS and lysed in IP lysis buffer (50 mM Tris-HCl, pH 7.4; 150 mM NaCl; 2 mM EDTA; 0.5 mM EGTA; 1% Triton X-100; 10% glycerol; protease and phosphatase inhibitor cocktail; 1 µM TSA; 20 mM nicotinamide; 1 mM dithiothreitol) with brief sonication. Protein A/G magnetic beads (order number 88,803 from Thermo Fisher Scientific, Waltham, MA, USA) were washed three times with IP lysis buffer in low retention tubes (Kisker Biotech, Steinfurt, Germany) before adding 1 mg protein (per 25 µL beads), followed by 1 h incubation at 4 °C while rotating. Afterwards, the beads were removed and the samples were incubated on a tube rotator at 4 °C overnight with 1 µg rabbit anti-LC3A (Cell Signaling, Danvers, MA, USA; order number 4599), rabbit anti-LC3B (Sigma-Aldrich, St. Louis, MO, USA; order number L7543), or anti-LC3C (Abcam, Cambridge, UK; order number ab150367). Thereafter, magnetic beads reconstituted in IP lysis buffer were applied to the samples and incubated for 1 h on a tube rotator at 4 °C. The beads were washed five times with IP washing buffer (50 mM Tris-HCl, pH 7.4; 200 mM NaCl; 2 mM EDTA; 0.5 mM EGTA; 1% Triton X-100; protease and phosphatase inhibitor cocktail; 1 µM TSA; 20 mM nicotinamide; 1 mM dithiothreitol). Then, the beads were stripped in loading buffer (150 mM Tris-HCl, pH 6.8; 15% glycerol; 10% mercaptoethanol; 3% SDS; 0.003% bromophenol blue) for 10 min on a thermomixer at 36 °C before brief centrifugation and bead removal. The isolated proteins were boiled for 5 min at 95 °C, loaded onto a 15% SDS gel and separated by standard PAGE-Western blotting. To quantify the immunoprecipitated LC3 proteins, the same antibodies were used as for the precipitation itself; acetylated lysines were visualized with rabbit anti-acetylated lysine antibody (1:1000, Cell Signaling, Danvers, MA, USA; order number 9441).

### 2.5. Immunocytochemistry

Primary human fibroblasts (IMR90 cells) were grown on glass cover slips, treated as appropriate, and fixed with 4% paraformaldehyde. Unspecific epitopes were blocked with 3% BSA before permeabilization with 0.1% Triton X-100 in PBS. The fixed cells were then incubated overnight with primary antibodies diluted in PBS containing 1% BSA: rabbit anti-LC3A (1:200, from Cell Signaling, Danvers, MA, USA; order number 4599), mouse anti-LC3B (1:200, from NanoTools, Teningen, Germany; order number 0260-100), rabbit anti-LC3C (1:200, from Cell Signaling, Danvers, MA, USA; order number 14736), guinea pig anti-p62 (1:400, from Progen, Heidelberg, Germany; order number GP62-C), rabbit anti-LAMP2 (1:1000, from BioCat, Heidelberg, Germany; order number WA-ABV10697.100). The cells were subsequently incubated with Cy2-, Cy3- and Cy5-coupled secondary antibodies (1:500, from Jackson Immunoresearch, West Grove, PA, USA) for 2 h at room temperature. Cell nuclei were counterstained with 1 µg/mL 4,6-diamidino-2-phenylindole (DAPI, from Sigma-Aldrich, St. Louis, MO, USA). The cells were then analyzed and photographed using a confocal laser-scanning microscope (TCS SP5 from Leica Microsystems, Wetzlar, Germany). All pictures were taken with a 63x objective and 2x zoom at the highest possible resolution (2048 × 2048 pixels). 

### 2.6. Subcellular Fractionation

IMR90 cells were cultivated on 10 cm dishes, treated as described, and lysed in 500 µL fractionation buffer (20 mM HEPES, pH 7.4; 250 mM sucrose; 10 mM KCl; 1.5 mM MgCl_2_; 1 mM EDTA; 1 mM EGTA; protease inhibitor cocktail (Roche, Basel, Switzerland)). The lysates were then passed 10 times through a 25-gauge needle using a 1 mL syringe and left on ice for 20 min. Nuclei were isolated through centrifugation at 720 g for 10 min at 4 °C. The supernatant was removed and centrifuged again at 10,000 g for 10 min at 4 °C. The pellet discarded, and 2% SDS were added to the cytosolic supernatant. The nuclear fraction was washed by adding 500 µL fresh fractionation buffer and repeating the steps described above. The pellet was then reconstituted in 100 µL fractionation buffer with 2% SDS. Protein concentration was determined using a BCA kit (Thermo Fisher Scientific, Waltham, MA, USA) as per the manufacturer’s protocol. An amount of 10 μg protein was loaded on a 15% SDS gel and separated by electrophoresis. The proteins were transferred onto a nitrocellulose membrane, blocked with 4% non-fat milk in PBST, and detected with the following primary antibodies: rabbit anti-LC3A (1:1000, Cell Signaling, Danvers, MA, USA; order number 4599), rabbit anti-LC3B (1:1000, Sigma-Aldrich, St. Louis, MO, USA; order number L7543), rabbit anti-LC3C (1:1000, Abcam, Cambridge, UK; order number ab150367), mouse anti-histone H3 (1:1000, Cell Signaling, Danvers, MA, USA; order number 14269), mouse anti-α-tubulin (1:1000, Sigma-Aldrich, St. Louis, MO, USA; order number T9026). The detection by secondary antibodies was done as described in Section 2.2. 

### 2.7. Knock-Down Experiments

IMR90 cells were transfected by electroporation (Nucleoffector 2b, Lonza, Basel, Switzerland; program: U-24). A number of 8000 cells per transfection were reconstituted in 400 µL electroporation buffer (10 mM HEPES, pH 7.8; 135 mM KCl; 2 mM MgCl_2_; 200 µM CaCl_2_; 2 mM EGTA; 25% FCS) with 30 µg siRNA (small interfering RNA) or scrRNA (scrambled RNA) and transferred to an electroporation cuvette (Sigma-Aldrich, St. Louis, MO, USA; order number Z706094-50EA). After electroporation, the cells were left for 8 min at 37 °C in an incubator containing 5% CO_2_. Thereafter, 600 µL culture medium was added, and the cells were seeded in a 24-well plate with coverslips adding an additional 1000 µL culture medium. One day later, the cells were treated with 100 nM Ex527 or vehicle (DMSO). After 24 h, the medium was replaced, and the cells harvested by fixation after an additional 24 h. Four hours prior to fixation, the cells were starved for 4 h using Earle’s balanced salt solution and/or treated with 2 µM BafA1 where appropriate.

### 2.8. Bioinformatic Analysis

Protein sequences from *H. sapiens* LC3A, LC3B, LC3C, GABARAP, GABARAP-L1, GABARAP-L2 and were obtained from Ensembl [14] and aligned using Clustal Omega from EMBL-EBI. A phylogenetic tree based on DNA data from Ensembl was also constructed with Clustal Omega from EMBL-EBI and the Interactive Tree Of Life software [15] to incorporate *H. sapiens* LC3A, LC3B, LC3C, GABARAP, GABARAP-L1, GABARAP-L2; *D. melanogaster* ATG8A and ATG8B; and *S. cerevisiae* ATG8 as out-group. ATG7 binding sites and lipidation sites were extracted from previous studies revealing their exact amino acid sequences [16,17]. Specific interaction partners of human Atg8 family proteins were assembled from previously published data [18]. Their sequences were then retrieved from Ensembl and analyzed in the following manner: the protein sequences of interaction partners unique to a specific ATG8 were analyzed for putative LIR domains that would follow the established LIR sequence pattern: Y/F/W–X–X–V/I/L (X = any amino acid) [19]. Finally, the relative occurrence of all amino acids on the four LIR positions was calculated.

### 2.9. Statistical Analysis 

Depending on the structure of the data, either one-way or two-way analysis of variance (ANOVA) was performed, followed by Benjamini and Hochberg adjustment for multiple comparisons. In the case of two-way ANOVA analysis, variable #1 was usually the presence or absence of TSA or Ex527, while variable #2 was the identity of the LC3s to be compared. Statistical evaluation of the autophagic fluxes was performed by Kruskal-Wallis one-way ANOVA (on Ranks) since the data did not always pass the normality test implemented in the employed software GraphPad Prism 7.03. Post-hoc test significances with smaller *p*-values than 0.05 are indicated by asterisks or hash signs in the figures. Quantitative results are generally presented as arithmetic mean and standard deviation (SD).

## 3. Results

### 3.1. Deacetylase Inhibition Selectively Impairs LC3B Functionality, but not LC3A or LC3C

Bafilomycin A1 (BafA1) is a lysosomal vacuolar H^+^-ATPase (V-ATPase) inhibitor that blocks autophagosome–lysosome fusion and the acidification of autophagolysosomes. This leads to a compensatory translocation of LC3B-I from the nucleus into the cytoplasm in a SIRT1-dependent manner [10]. The cytoplasmic LC3B-I is then phosphatidylethanolamine-conjugated (“lipidated”) and thereby converted to functional LC3B-II, the membrane-associated form of the protein. LC3B-II is important for the elongation of the phagophore into autophagosomes, which may be a shared property of all six members of the mammalian ATG8 family [11,12,20]. LC3B-II stays attached to the newly formed autophagosome, but it is degraded after their fusion with lysosomes or recycled by delipidation through cytosolic ATG4. The role of LC3B during autophagy has been well studied and described; however, only little is known about the regulation and cellular localization of the other LC3 family members, LC3A and LC3C. In the following, we wanted to investigate whether LC3A and LC3C might be functional analogues to LC3B that may compensate for a loss of functional LC3B. Hence, LC3B was functionally decapacitated by different means, and the response of LC3A and LC3C was monitored. 

LC3B decapacitation was first induced pharmacologically by two inhibitors of protein deacetylation: Ex527, a SIRT1 inhibitor, which should lead to a nuclear trapping of LC3B [9,10], and trichostatin A (TSA), a broad-spectrum inhibitor of non-sirtuin histone deacetylases (HDACs). The effects of these agents on LC3 expression and lipidation with or without BafA1 were studied by Western blotting.

**Figure 1:**
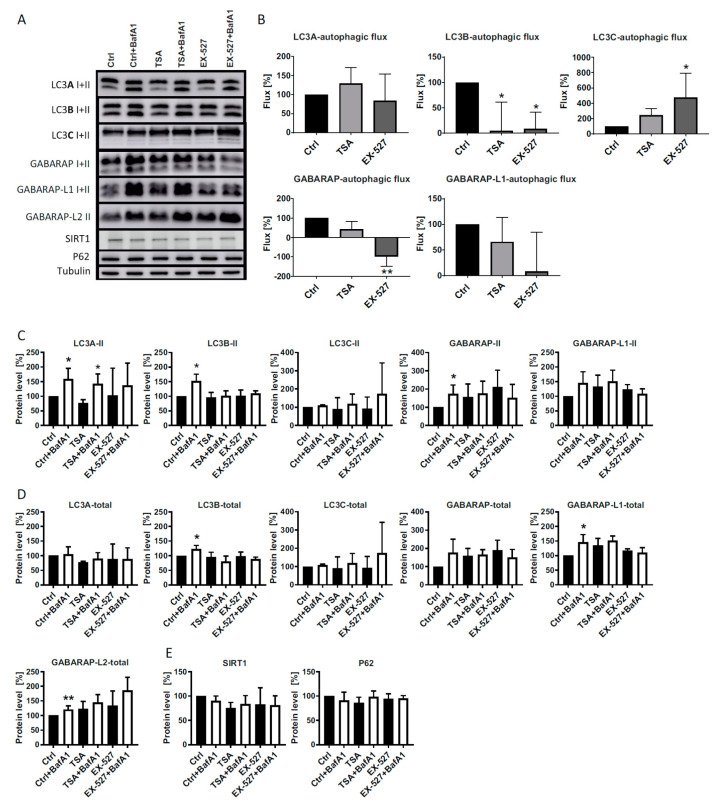
Analysis of LC3 family protein expression and lipidation. (**A**) Total cell lysates from primary human fibroblasts treated with BafA1, Ex527 or TSA for 48h were analyzed by Western blot as indicated. (**B**–**E**) Densitometric quantifications of the immunoreactive bands from *n* = 3 independent experiments. Raw numbers were first normalized to tubulin, and the resulting values were related to the similarly tubulin-normalized control (Ctrl). (**B**) Autophagic flux analyses in cells treated as indicated for 48h. Analysis was performed for each ATG8 protein by determining the difference, between BafA1-treated and BafA1-untreated cells, of the intensity of the lipidated bands running below the unlipidated bands [Flux = (LC3II/Tub)_with BafA1_—(LC3II/Tub)_without BafA1_]. Significant changes (by one-way ANOVA on ranks) versus the control: * *p* ≤ 0.05; ** *p* ≤ 0.01. (**C**) Raw intensities of all lipidated protein bands (tubulin-normalized). Significant changes (by two-way ANOVA) versus BafA1-untreated cells: * *p* ≤ 0.05; ** *p* ≤ 0.01. (**D**) Raw intensities of all lipidated and unlipidated protein bands (tubulin-normalized). Statistical analysis was done as in (**C**). (**E**) Quantification of SIRT1 and p62 expression (tubulin-normalized).

LC3A was substantially lipidated (in terms of the lower LC3A-II band) at baseline and lipidation accrued with BafA1, but the BafA1 effect, a widely used proxy for autophagic flux, was not modulated by Ex527 or TSA treatment (Figure 1A,B). Hence, LC3A did not respond to deacetylase inhibition nor to induced LC3B trapping (see below), indicating that LC3A could keep its functional capacity even when LC3B was trapped in the nucleus. Regarding LC3B, this protein lost its role for cytosolic autophagy as expected from its trapping in the nucleus (Figure 1B). LC3C, in contrast, became relatively more lipidated with Ex527 or TSA treatment as would be consistent with a compensatory response model in which LC3C would partially replace LC3B. However, LC3C’s lipidation at baseline and its response to BafA1 were rather low, making a complete functional replacement of LC3B by LC3C unlikely (Figure 1A,B). Regarding the two GABARAPs featuring a lipidated band, these proteins behaved similarly as LC3B in largely losing their response to BafA1 under Ex527 or TSA treatment. Hence, compensation or merely buffering of LC3B loss through GABARAP and GABARAP-L1 is unlikely (Figure 1A,B). GABARAP-L2 did not reveal any lipidated form that would have been distinguishable by its running behavior in SDS gels (Figure 1A).

To track the origins of the calculated, differential induction of lipidation (Figure 1B) that reflects autophagic flux, the lipidated forms of all LC3 proteins were inspected separately (Figure 1C), as was their total protein expression (Figure 1D). A significant effect of BafA1 on LC3 lipidation was seen for LC3A, LC3B and GABARAP under baseline conditions, and for LC3A under TSA treatment. Notably, the effect of BafA1 on LC3B lipidation was fully abolished by TSA and Ex527 treatment, as expected. The lack of effect of BafA1 on GABARAP lipidation under TSA and Ex527 treatment was apparently attributable to an increase in the unlipidated form of this protein (Figure 1C,D). However, this increase did not reach statistical significance, similarly as all other differences in lipidated or total LC3 protein expression induced by TSA and Ex527. Hence, none of the observed differences evoked by the deacetylase inhibitors were explicable by off-target effects on protein transcription or translation. Moreover, Ex527 and TSA also did not modulate the protein levels of SIRT1 or p62 (Figure 1E).

**Figure 2:**
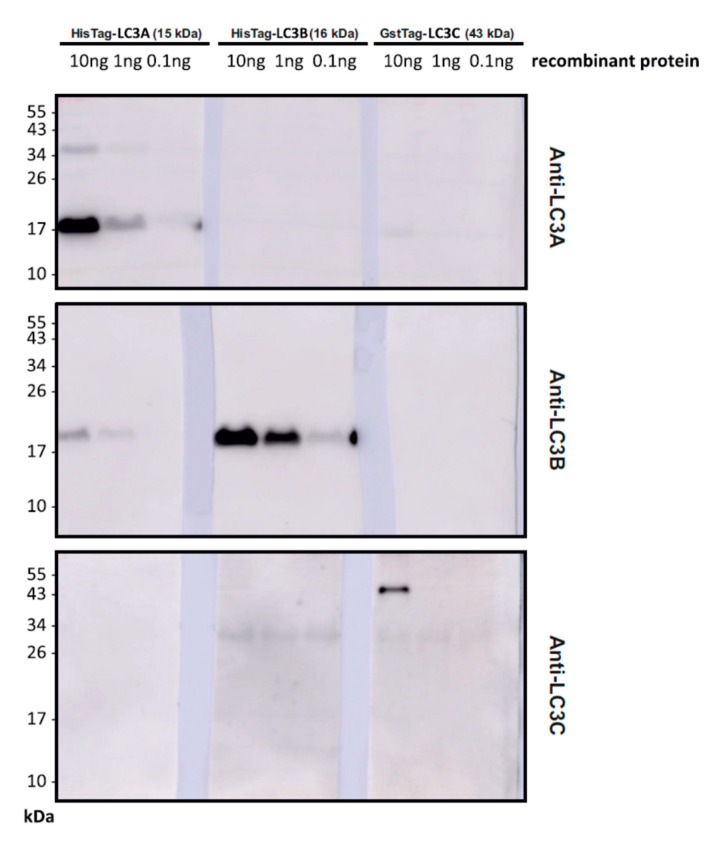
Western blot analysis of anti-LC3 antibody specificity. Each framed box represents three membrane pieces (LC3A/LC3B/LC3C) that were developed with the indicated antibody (right margin) simultaneously. Membrane pieces for each LC3 had been cut from the same master membrane loaded several times with only one of the LC3s to assure equal transfer efficiency. The loaded amount per lane and the expected size (in kDa) are indicated. The employed antibodies were: anti-LC3A from Cell Signaling (#4599), anti-LC3B from Sigma (#L7543), anti-LC3C from Abcam (#ab150367).

To ascertain the specificity of the antibodies employed in the Western blots analyzed above, a series of control blots was performed using pure, untagged or tagged, recombinant LC3A, LC3B and LC3C proteins as antigens. The three antibodies were found to exhibit excellent specificity for their respective targets (Figure 2). The strongest observed cross-reactivity was elicited by the anti-LC3B antibody against recombinant LC3A, which was nevertheless recognized with only about 1–2% relative efficacy.

### 3.2. Deacetylase Inhibition Traps LC3B in the Nucleus, but Only Mildly Affects LC3A and Mobilizes LC3C

**Figure 3:**
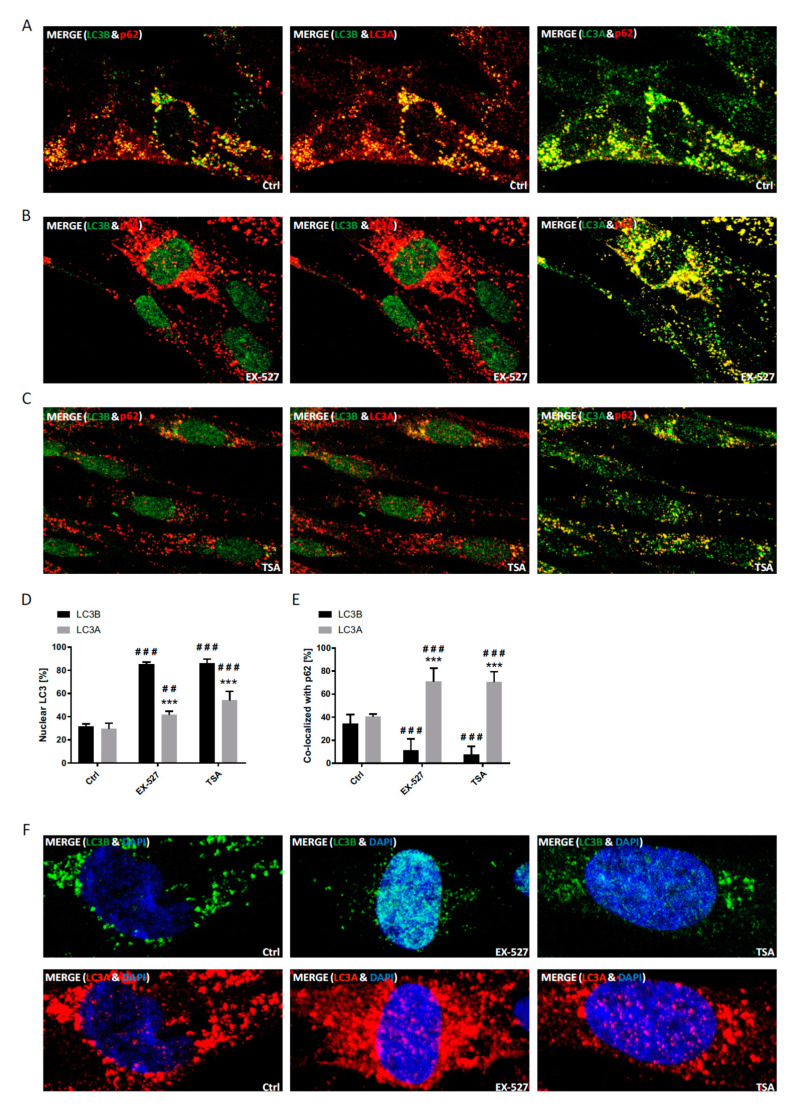
Analysis of the cellular localization of LC3A and LC3B in response to pharmacological sirtuin inhibition. Primary human fibroblasts treated with BafA1, Ex527 or TSA as indicated were immunostained with LC3A, LC3B and p62 antibodies and analyzed microscopically. (**A**) Cellular localization of LC3A, LC3B and p62 after treatment with BafA1 alone. (**B**) Cellular localization of LC3A, LC3B and p62 after treatment with Ex527 and BafA1. (**C**) Cellular localization of LC3A, LC3B and p62 after treatment with TSA and BafA1. (**D**) Image analytical quantification of the relative nuclear fraction of LC3A and LC3B in cells treated as indicated and counterstained with DAPI. Significant changes (by two-way ANOVA) versus the control: ^##^ *p* ≤ 0.01; ^###^ *p* ≤ 0.001; significant changes between the LC3s: *** *p* ≤ 0.001. (**E**) Relative co-localization of LC3A and LC3B with p62 quantified by image analysis. Statistical analysis was done as in D. Data in (**D**) and (**E**) were derived from three photographed, independent experiments (*n* = 3), in which 10–50 cells per image were analyzed. (**F**) Magnified images of cells immunostained for LC3A or LC3B including the chromatin counterstain (DAPI).

**Figure 4:**
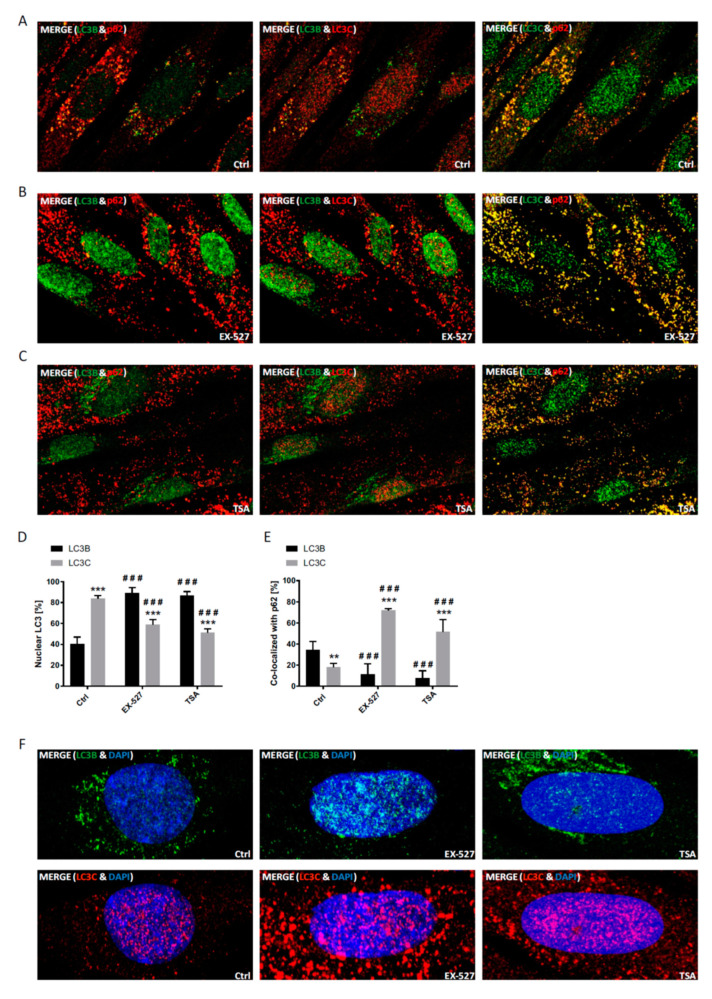
Analysis of the cellular localization of LC3C and LC3B in response to pharmacological sirtuin inhibition. Primary human fibroblasts were treated with BafA1, Ex527 or TSA and analyzed as in Figure 3. (**A**) Cellular localization of LC3C, LC3B and p62 after treatment with BafA1 alone. (**B**) Cellular localization of LC3C, LC3B and p62 after treatment with Ex527 and BafA1. (**C**) Cellular localization of LC3C, LC3B and p62 after treatment with TSA and BafA1. (**D**) Quantification of the relative nuclear fraction of LC3C and LC3B in cells treated as indicated. Significant changes (by two-way ANOVA) versus the control: ^###^ *p* ≤ 0.001; significant changes between the LC3s: ** *p* ≤ 0.01; *** *p* ≤ 0.001. (**E**) Relative co-localization of LC3C and LC3B with p62 quantified by image analysis. Statistical analysis was done as in D. Data in (**D**) and (**E**) were derived from three photographed, independent experiments (*n* = 3), in which 12–45 cells per image were analyzed. (**F**) Magnified images of cells immunostained for LC3C or LC3B including the chromatin counterstain (DAPI).

As a functional involvement of the LC3 proteins in canonical autophagy is only conceivable in the cytosol (where lipidation usually takes place), the subcellular distribution of LC3A, LC3B, LC3C was investigated by confocal fluorescence microscopy. Two main parameters were quantified: First, the nuclear fraction of each LC, indicative of functional decapacitation; and second, the degree of co-localization with p62, a ubiquitin and autophagy receptor, in the cytosol. The latter co-localization usually reflects the recruitment of the LC3 protein to the site of autophagy. In the absence of BafA1, p62 and LC3A mainly localized to the cytoplasm, whereas LC3B was mostly found in the nucleus, while LC3C appeared to be present in both nucleus and cytoplasm (Supplementary Figure S1). Upon late 4 h BafA1 treatment, however, p62 was visibly reorganized and probably oligomerized in vesicular structures in the cytoplasm, indicative of autophagosome formation (Figure 3 and Figure 4, Supplementary Figure S2). The increased p62 immunofluorescence signal with BafA1, especially together with TSA or Ex527 (Supplementary Figure S2), despite evidently unchanged total protein expression (Figure 1E), supports the idea of a local accumulation and activation of p62 in topologically compact vesicular structures which led to a better quantitative detection of this protein than in dispersed form. For the above reasons, the subsequent detailed analyses of LC3 intracellular distribution were generally conducted with late BafA1 treatment. Through the BafA1-effectuated inhibition of the later steps of autophagy, substrate accumulation would occur, and early autophagosome formation would be compensatorily induced [6].

The nuclear fraction of LC3A and its degree of co-localization with p62 were analyzed by differential staining against LC3B under normal and LC3B decapacitation conditions (Figure 3). LC3A and LC3B were both about 30–40% localized to the nucleus at baseline. Treatment with TSA and Ex527 led to a massive rise in the nuclear fraction of LC3B as expected, but only to a moderate rise in the nuclear fraction of LC3A (Figure 3D). Correspondingly, the about equal co-localization of both proteins with p62 at baseline dropped for LC3B, but significantly rose for LC3A (Figure 3E). These observations are compatible with, and may even suggest, a functional compensation of LC3B loss through LC3A.

The nuclear fraction of LC3C and its degree of co-localization with p62 was analyzed accordingly (Figure 4). At baseline, LC3C was already mostly restricted to the nucleus. Treatment with TSA and Ex527; however, led to a highly significant translocation into the cytosol, i.e., in the opposite direction of LC3B (Figure 4D). Correspondingly, the co-localization of LC3C with p62 approximately tripled, whereas LC3B’s co-localization with p62 in the same cells plummeted as before (Figure 4E). Both shifts again indicate a converse behavior of LC3B and LC3C that may be compensatory.

It is notable that in the experiments before, the effects of TSA and Ex527 appeared to be very similar, with the exception of a moderately differential induction of lipidation of LC3C and GABARAP (Figure 1B). Beyond this difference, TSA led to an apparently more perinuclear accumulation of LC3B, whereas Ex527 rather kept LC3B deeper inside the nucleus (Figure 4F). This indicates that the complete egress and functional activation of LC3B in the cytoplasm may be dependent on more or other deacetylases than sirtuins alone. In fact, it has been shown that HDAC family members inhibited by TSA are required for the functional activation of LC3B-II in Hela cells subjected to serum deprivation [21]. Under these premises, it appears likely that the increased presence of LC3C in the cytosol (Figure 4D) is indeed a compensatory response to LC3B decapacitation rather than an off-target, direct consequence of Ex527 treatment. At the employed concentrations of 10 nM TSA (reported IC_50_ value 1–100 nM) and 100 nM Ex527 (reported IC_50_ value 60–100 nM), both inhibitors should be exclusively affecting their respective deacetylase targets [22,23]. We also attempted to verify the specificity of the effects of the deacetylase inhibitors TSA and Ex527 by monitoring LC3 acetylation. Therefore, cell lysates were immunoprecipitated with highly specific antibodies (Figure 2) against LC3A, LC3B and LC3C, and the recovered proteins were probed for global lysine acetylation (Supplementary Figure S3). No relevant differences could be detected by this approach, which might find its explanation in the fact that the employed anti-acetyl lysine epitope antibody may well require a certain sequence context for efficient binding. Moreover, LC3 proteins are predicted to contain several potential sites of acetylation, which might result in high background acetylation interfering with the detection of changes on individual regulatory lysines. 

**Figure 5:**
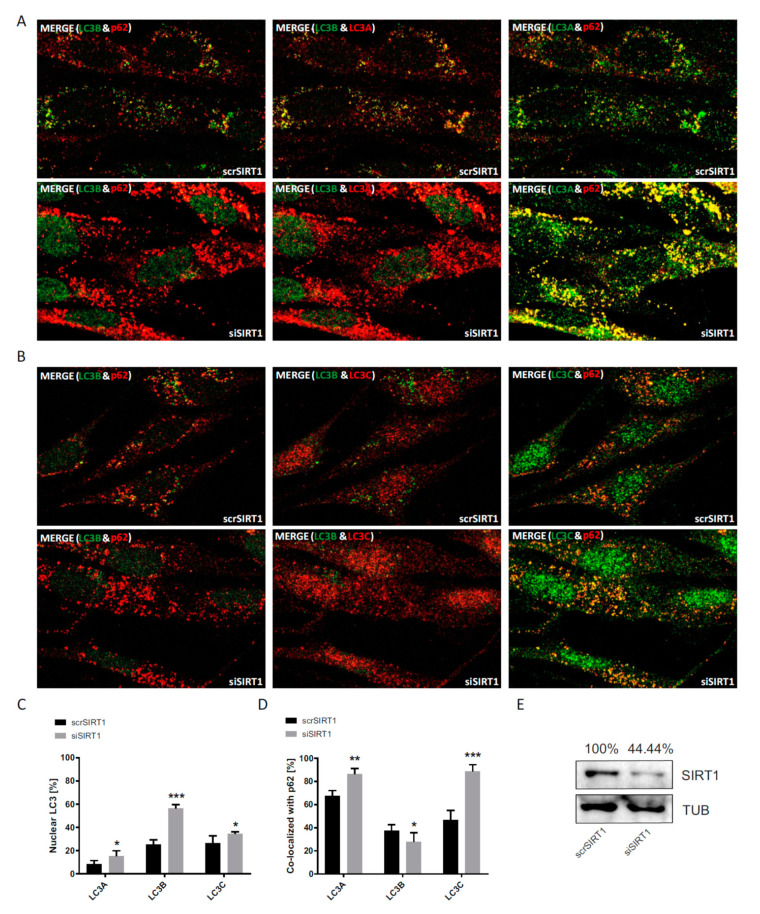
Analysis of the cellular localization of LC3A, LC3B and LC3C in response to siRNA-mediated Sirtuin1 knock-down. (**A**) Cellular localization of LC3A, LC3B and p62 after transfection with Sirtuin1 siRNA (siSIRT1-RNA) or scrambled RNA (scrSIRT1-RNA) under BafA1 treatment. (**B**) Cellular localization of LC3C, LC3B and p62 after transfection with Sirtuin1 siRNA (siSIRT1-RNA) or scrambled RNA (scrSIRT1-RNA) under BafA1 treatment. (**C**) Quantification of the relative nuclear fraction of LC3A, LC3B and LC3C in cells transfected as indicated. (**D**) Relative co-localization of LC3A, LC3B and LC3C with p62 quantified by image analysis. Significant changes (by two-way ANOVA) versus scrSIRT1-treated cells: * *p* ≤ 0.05; ** *p* ≤ 0.01; *** *p* ≤ 0.001. Data in (**C**) and (**D**) were derived from three photographed, independent experiments (*n* = 3), in which 12–45 cells per image were analyzed. (**E**) Sirtuin1 protein expression after transfection with scrSIRT1-RNA or siSIRT1-RNA. Tubulin was used as loading control.

To complement the above pharmacological strategy of sirtuin inhibition by an independent, second approach, sirtuin 1 knock-down experiments were performed (Figure 5). To this end, primary human fibroblasts were transfected by electroporation with either Sirtuin1 siRNA (siSIRT1-RNA) or scrambled RNA (scrSIRT1-RNA) as control. In these experiments, all LC3 proteins were less restricted to the nucleus than under physiological control conditions even when transfected only with scrambled RNA (Figure 5A–C). Thus, electroporation may have led to a persistent impairment of the nuclear barrier, indicating that all nuclear localization results should be read with care. Inspecting the difference in nuclear localization after siSIRT1-RNA versus scrSIRT1-RNA treatment, LC3B was selectively more trapped in the nucleus than LC3A and LC3C (Figure 5C). More importantly, cytosolic LC3A and LC3C both showed an increased co-localization with p62 after siSIRT1-RNA treatment, whereas LC3B’s co-localization was mildly reduced (Figure 5D). These data parallel the results from pharmacological sirtuin inhibition and again point to a potential functional complementation of LC3B by the other LC3 family members.

### 3.3. Distribution and Redistribution of the LC3s under Conditions of Starvation

**Figure 6:**
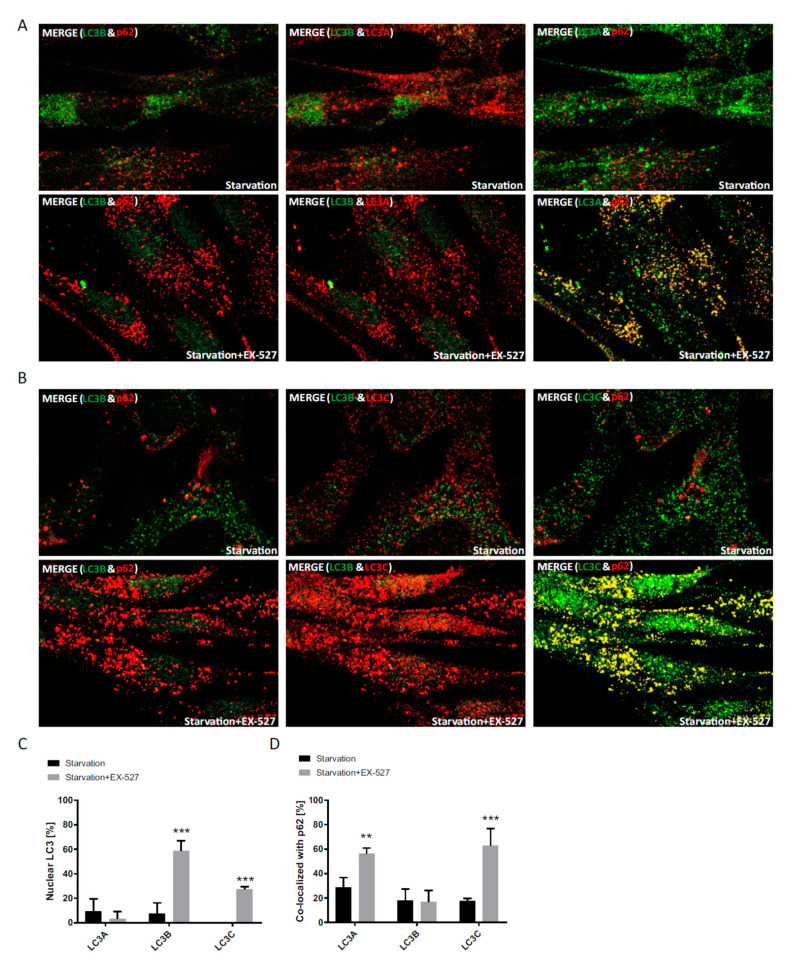
Analysis of the cellular localization of LC3A, LC3B and LC3C in response to pharmacological sirtuin inhibition under starvation conditions. Primary human fibroblasts were grown under starvation conditions and treated with BafA1 and Ex527. (**A**) Cellular localization of LC3A, LC3B and p62 after treatment with Ex527 and BafA1. (**B**) Cellular localization of LC3C, LC3B and p62 after treatment with Ex527 and BafA1. (**C**) Quantification of the relative nuclear fraction of LC3A, LC3B and LC3C in cells treated as indicated. (**D**) Relative co-localization of LC3C, LC3B and LC3A with p62 quantified by image analysis. Significant changes (by two-way ANOVA) versus starvation-only treated cells: ** *p* ≤ 0.01; *** *p* ≤ 0.001. Data in (**C**) and (**D**) were derived from three photographed, independent experiments (*n* = 3), in which 12–45 cells per image were analyzed.

The behavior of LC3A, LC3B and LC3C in primary human fibroblasts was further studied under autophagy-inducing conditions of nutritional starvation. For reasons of comparability, the late 4 h BafA1 treatment was maintained. All LC3s were basically absent from the nucleus under these conditions, as was to be expected (Figure 6). Ex527 treatment led to a significant trapping of LC3B (by 60%) in the nucleus, but also LC3C was relocated to the nucleus (by 30%), while LC3A was unaffected (Figure 6C). Regarding p62, Ex527 treatment led to a pronounced increase in LC3A and LC3C co-localization with this protein, whereas LC3B was numerically unaffected owing to a higher association of the remainder cytosolic LC3B protein with p62 (Figure 6D). Thus, starvation had a major effect on the total nuclear fraction of all LC3 proteins (compare with the appropriate unstarved control experiments in Figure 3D and Figure 4D), whereas the relative cytosolic association of these proteins with p62 at baseline and under Ex527 were similar as under nourished conditions (compare with the unstarved experiments in Figure 3E and Figure 4E).

**Figure 7:**
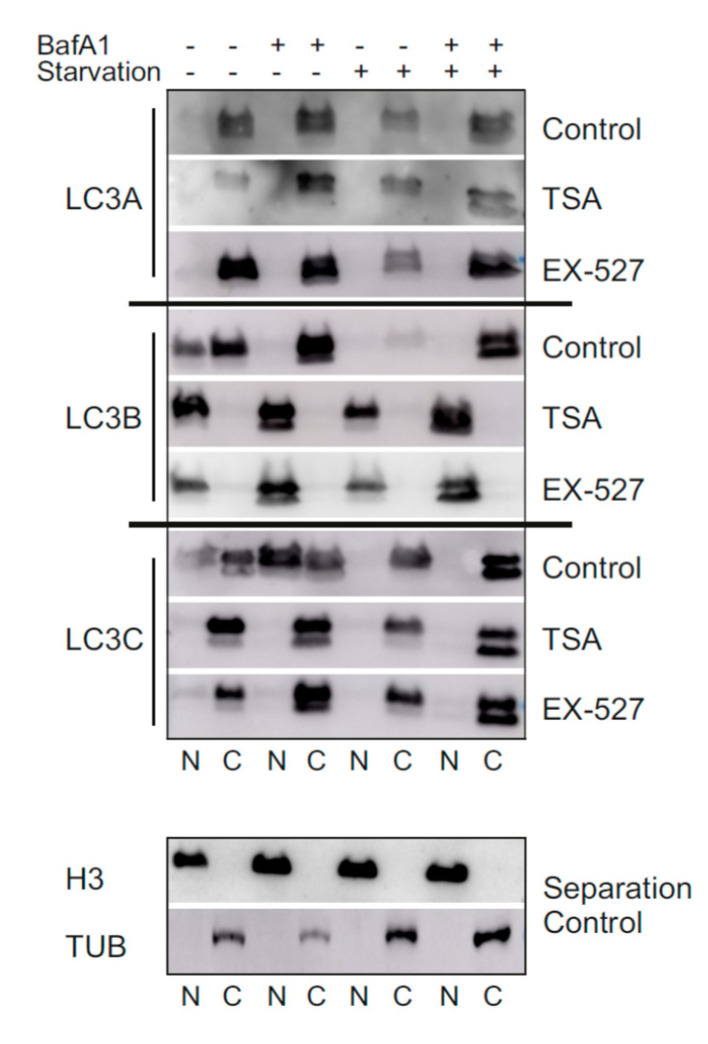
Western blot analysis of the cellular localization of LC3A, LC3B and LC3C in response to pharmacological sirtuin inhibition under normal and starvation conditions. Primary human fibroblasts were grown under normal or starvation conditions and were treated with BafA1, Ex527 or TSA, as indicated. Nuclear and cytosolic fractions of the cells were prepared and investigated for their protein content of LC3A, LC3B and LC3C. The nuclear marker histone H3 and the cytosolic marker tubulin were analyzed in selected fractions for general separation quality control purposes.

We aimed at an independent validation of the above result by a second method, namely Western blotting after subcellular fractionation. Nuclear and cytosolic fractions were prepared from cells treated with or without BafA1, Ex527 and TSA under normal or starvation conditions; the results are provided in Figure 7. Under normal, BafA1-positive conditions, LC3A and LC3B were mostly localized to the cytosol, whereas LC3C was more prominent in the nucleus. Starvation led all proteins to stay more sharply in the cytosol. TSA and Ex527, however, induced a complete shift of only LC3B into the nuclear fraction, irrespective of the boundary conditions. In LC3C, conversely, TSA and Ex527 treatment resulted in a sharp redistribution into the cytosol, even more so than anticipated from Figure 4D. In summary, the distributions and shifts in distribution derived from confocal image analysis (Figure 3, Figure 4 and Figure 6) were surprisingly well recapitulated in the Western blot analysis, supporting the notion of a functional buffering or even compensation of LC3B loss by LC3A and LC3C, respectively. The otherwise most relevant difference, a slightly higher cytosolic signal than expected from image quantification, was noted throughout all proteins and treatments and probably has a technical origin

### 3.4. Individual Knock-Downs of LC3A, LC3B and LC3C Lead to Selective Shifts in Their Congeners’ Subcellular Association

**Figure 8:**
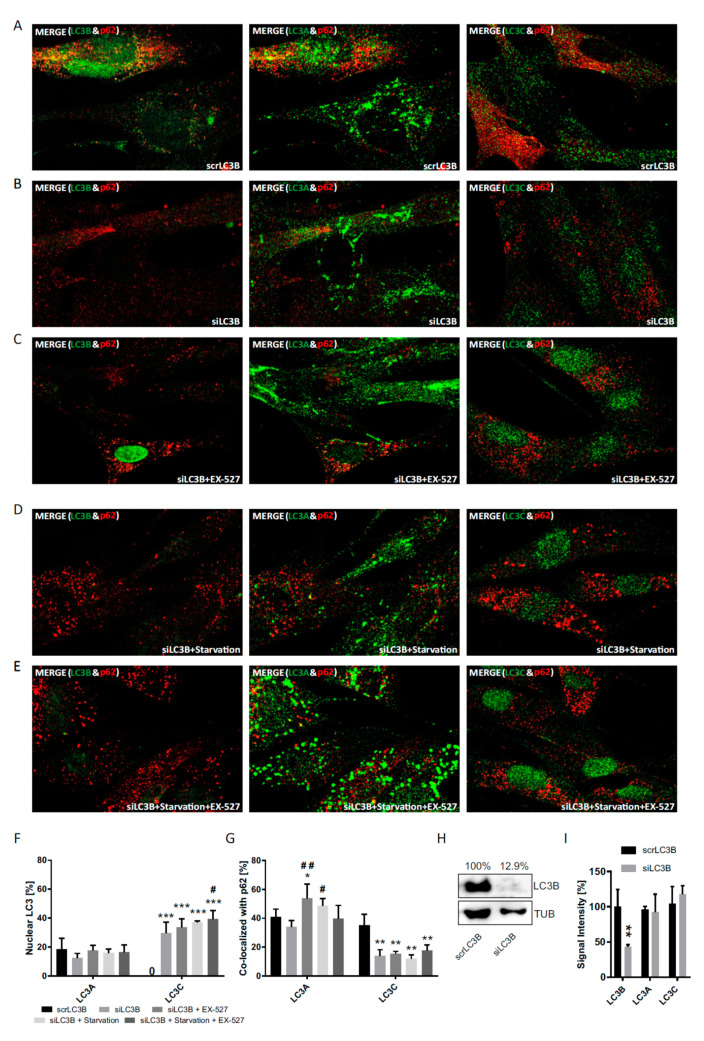
Analysis of the cellular localization of LC3A, LC3B, LC3C after siRNA-mediated LC3B knock-down. Primary human fibroblasts were treated with BafA1 after transfection with (**A**) scrLC3B-RNA, (**B**) siLC3B-RNA, (**C**) siLC3B-RNA and Ex527 treatment, (**D**) siLC3B-RNA and starvation, and (**E**) siLC3B-RNA and starvation and Ex527 treatment. (**F**) Quantification of the relative nuclear fraction of LC3A and LC3C in cells treated as indicated. (**G**) Relative co-localization of LC3A and LC3C with p62 quantified by image analysis. Significant changes (by two-way ANOVA) versus scrLC3B: * *p* ≤ 0.05; ** *p* ≤ 0.01; *** *p* ≤ 0.001; significant changes versus siLC3B: ^#^ *p* ≤ 0.05; ^##^ *p* ≤ 0.01. (**H**) LC3B protein expression in cells transfected with scrLC3B-RNA or siLC3B-RNA. Tubulin was used as loading control. (**I**) Control of knock-down and antibody specificity under immunocytochemistry conditions. Whole-cell signal intensities were quantified and evaluated by two-way ANOVA. Data in (**F**), (**G**) and (**I**) were derived from three photographed, independent experiments (*n* = 3), in which 12–45 cells per image were analyzed.

**Figure 9:**
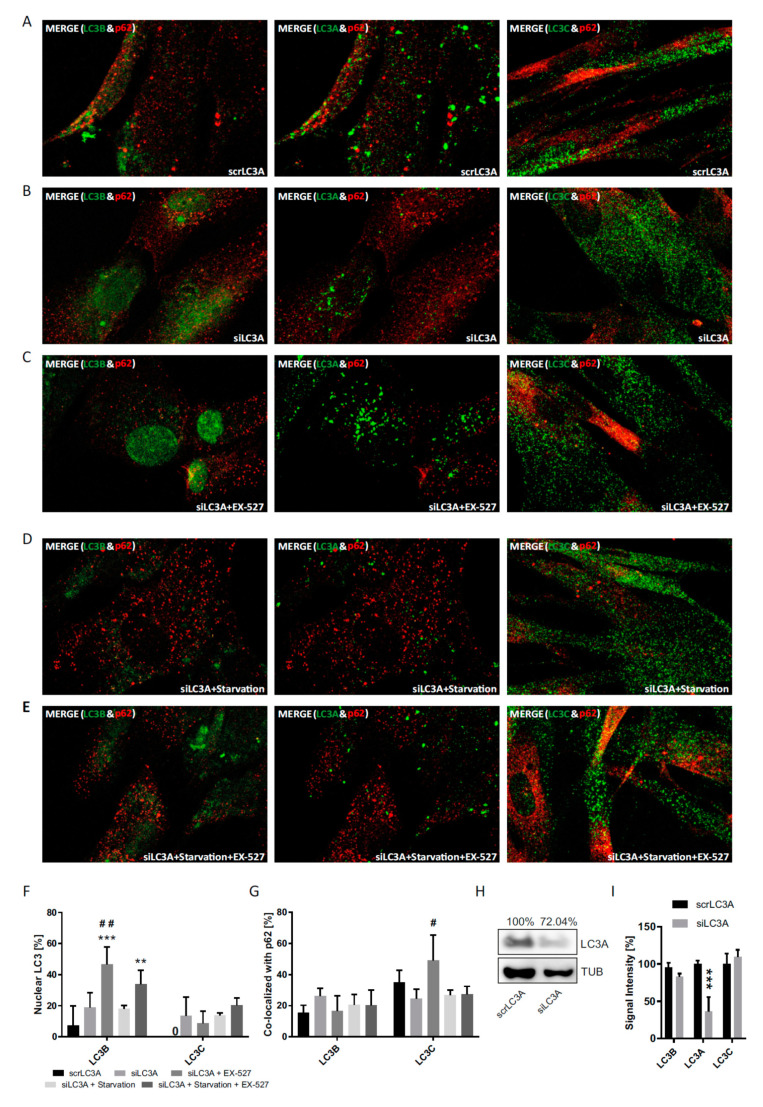
Analysis of the cellular localization of LC3A, LC3B, LC3C after siRNA-mediated LC3A knock-down. Primary human fibroblasts were treated with BafA1 after transfection with (**A**) scrLC3A-RNA and (**B**) siLC3A-RNA, **(C)** siLC3A-RNA and Ex527 treatment, (**D**) siLC3A-RNA and starvation, and (**E**) siLC3A-RNA and starvation and Ex527 treatment. (**F**) Quantification of the relative nuclear fraction of LC3B and LC3C in cells treated as indicated. (**G**) Relative co-localization of LC3B and LC3C with p62 quantified by image analysis. Significant changes (by two-way ANOVA) versus scrLC3A: ^**^ *p* ≤ 0.01; ^***^ *p* ≤ 0.001; significant changes versus siLC3A: ^#^ *p* ≤ 0.05; ^##^ *p* ≤ 0.01. (**H**) LC3A protein expression in cells transfected with scrLC3A-RNA or siLC3A-RNA. Tubulin was used as loading control. (**I**) Control of knock-down and antibody specificity under immunocytochemistry conditions. Whole-cell signal intensities were quantified and evaluated by two-way ANOVA. Data in (**F**), (**G**) and (**I**) were derived from three photographed, independent experiments (*n* = 3), in which 12–45 cells per image were analyzed.

**Figure 10:**
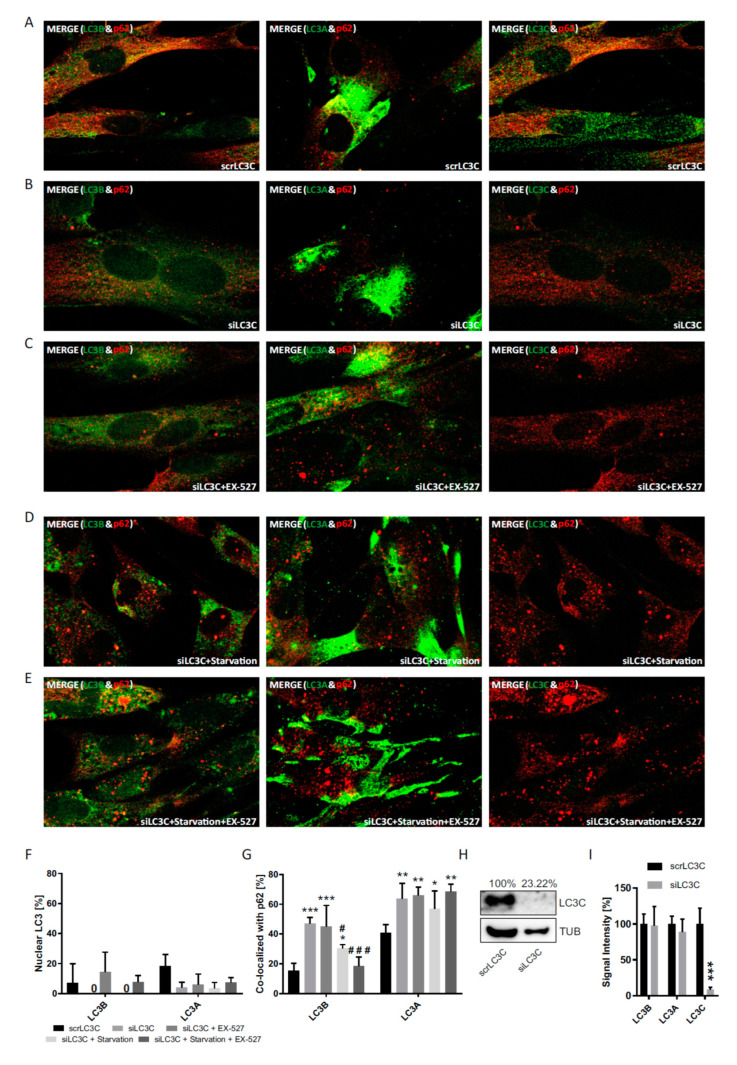
Analysis of the cellular localization of LC3A, LC3B, LC3C after siRNA-mediated LC3C knock-down. Primary human fibroblasts were treated with BafA1 after transfection with (**A**) scrLC3C-RNA, (**B**) siLC3C-RNA, (**C**) siLC3C-RNA and Ex527 treatment, (**D**) siLC3C-RNA and starvation, and (**E**) siLC3C-RNA, starvation and Ex527 treatment. (**F**) Quantification of the relative nuclear fraction of LC3A and LC3B in cells treated as indicated. (**G**) Relative co-localization of LC3A and LC3B with p62 quantified by image analysis. Significant changes (by two-way ANOVA) versus scrLC3C: * *p* ≤ 0.05; ** *p* ≤ 0.01; *** *p* ≤ 0.001; significant changes versus siLC3C: ^#^ *p* ≤ 0.05; ^###^ *p* ≤ 0.001. (**H**) LC3C protein expression in cells transfected with scrLC3C-RNA or siLC3C-RNA. Tubulin was used as loading control. (**I**) Control of knock-down and antibody specificity under immunocytochemistry conditions. Whole-cell signal intensities were quantified and evaluated by two-way ANOVA. Data in (**F**), (**G**) and (**I**) were derived from three images per treatment from three photographed, independent experiments (*n* = 3), in which 12–45 cells per image were analyzed.

To expand the so far applied strategies of LC3B cytosolic decapacitation by pharmacological treatment and sirtuin 1 suppression, individual knock-downs of LC3B, as well as LC3A and LC3C, were performed in primary human cells (Figure 8, Figure 9 and Figure 10). Notably, the total protein is affected by these knock-downs, whereas the pharmacological strategies only affected their intracellular distribution (Figure 3, Figure 4 and Figure 5) and left total protein levels unaltered (Figure 1D). A common result of these experiments was a very low nuclear fraction (<20%) of all LC3 proteins even when the cells had been transfected with scrambled RNAs (scrLC3A, scrLC3B, scrLC3C), indicating an impaired of the nuclear membrane due to electroporation as discussed above for scrSIRT1-RNA treatment. 

Nevertheless, one major, statistically significant effect could be observed with regard to the nucleus: authentic siLC3B-RNA treatment led to a stable increase in nuclear LC3C irrespective of the other conditions and was accompanied by a lower p62-association of LC3C in the cytosol (Figure 8). Thus, a loss of LC3B in the nucleus caused LC3C to travel into this compartment, precisely the opposite to when LC3B was accumulated in the nucleus by Ex527 treatment, which caused LC3C to leave this compartment, to highly associate with p62 (Figure 4) and likely to engage in cytosolic autophagy (Figure 1B). In a functional sense, LC3C seems to always travel to where LC3B has been lost and where this loss is functionally most severe, be it the cytosol or the nucleus. This interpretation is supported by the established essential role of LC3 in the nucleus [24]. To further back this notion, even LC3B associated more intensely with p62 in the cytosol when LC3C was globally knocked down (Figure 10). Additionally, siLC3C-RNA treatment also induced a moderate increase in the association of LC3A with p62 in the cytosol.

Regarding the effects of siLC3A-RNA treatment, this manipulation had only minor effects on the other LC3s. Their nuclear fractions were very low for technical reasons as mentioned, but Ex527 was still potent enough to induce LC3B nuclear trapping; other significant effects were not observed (Figure 9). Still, the efficiency of this knock-down was limited (Figure 9H). In summary, LC3A was mostly unaffected by changes in activity (and particularly distribution) of the other LC3s, but became activated in the cytosol in terms of increased p62 association when necessary. In general, though, all LC3A changes were less pronounced than the mutual adaptive behavior of LC3B and LC3C, which may thus act as a functional couple, while LC3A may rather act as emergency backup system.

Finally, we attempted to induced compensatory distributional shifts in the LC3 pool by knocking down Lamp-2, the lysosome-associated membrane glycoprotein 2, which is a major glycoprotein of the lysosomal membrane and thus plays an important role in the final stage of autophagic protein degradation [4,8]. While the employed transfection strategy with siLamp2-RNA did result in the desired reduction of the protein by approximately 60% (Supplementary Figure S4), this manipulation resulted in a pronounced reduction of cell survival and a variable accumulation of debris in the surviving cells, such that an unbiased quantification was impossible. For reference, a formal quantification of selected survivor cells is provided in the supplement (Supplementary Figure S4).

### 3.5. Phylogenetic Tree Analysis and Binding Site Comparison Confirms a Common Ancestry of the LC3 and GABARAP Genes

**Figure 11:**
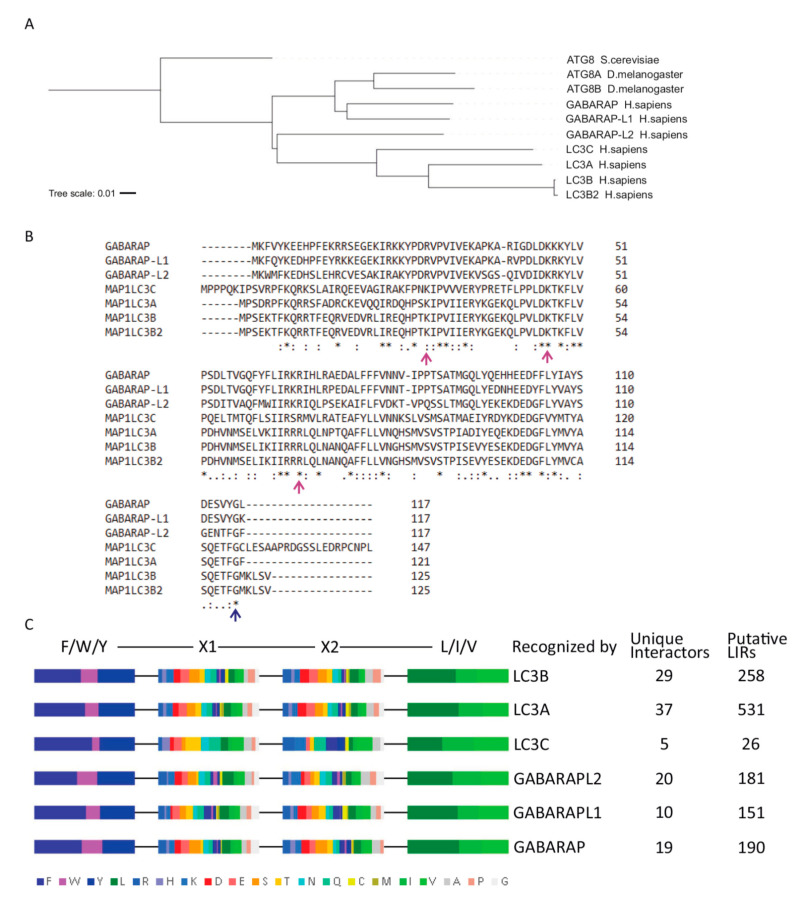
In silico sequence and binding partner analysis for the ATG8 protein family. (**A**) Phylogenetic tree of the ATG8 gene family featuring *H. sapiens* LC3A, LC3B, LC3B2, LC3C, GABARAP, GABARAP-L1, and GABARAP-L2, *D. melanogaster* ATG8A and ATG8B as well as *S. cerevisiae* ATG8. (**B**) Protein sequence alignment indicating identical (*), conserved (:) and semi-conserved (.) amino acids. Residues that have been found essential for ATG7 binding are indicated by a purple arrow, the conserved lipidation site glycine is marked with blue. (**C**) Meta-analysis of the amino acid composition of all putative (4-amino-acid) LIR motifs of the indicated number of recognized, exclusive ATG8 interactors as published [18]. The relative amino acid abundance for each position of a LIR motif was calculated and is shown by the indicated color-coding.

Since the above analyses revealed a differential deacetylase-dependent behavior of the structurally very similar proteins LC3A, LC3B and LC3C (Figure 11), we speculated that mammalian evolution could have involved a sub- or neo-functionalization of a common, ancient core function the different LC3 family members. To test this hypothesis, we performed a phylogenetic tree analysis and compared the conserved regions, binding sites, and recognized LIR motifs of the LC3 and GABARAP gene family members. The phylogenetic tree for *H. sapiens* LC3A, LC3B, LC3B2, LC3C, GABARAP, GABARAP-L1, GABARAP-L2, *D. melanogaster* ATG8, and *S. cerevisiae* ATG8 is shown in Figure 11A. *H. sapiens* LC3A and LC3B had a stronger relationship with each other than with LC3C, followed by GABARAP-L2, confirming previously published results [25]. Overall, the protein sequences of the *H. sapiens* LC3s and GABARAPs revealed a high degree of conservation, with 28 identical amino acids (AAs) and a total identity of 19%, as well as 48 similar amino acids. As expected, the LC3s were even more related and shared 43% identical AAs (with 63 identical AAs and 39 similar AAs). In particular, we observed substantial conservation of amino acids involved in ATG7 binding (R26, K49, R70 of LC3B) as well as universal conservation of the lipidation site (Figure 11B). This suggests that all human ATG8s retain the ability to interact with proteins necessary to catalyze autophagic processes [16].

To test for a selective targeting of the mammalian ATG8 proteins by autophagic receptors, a series of approximately 100 binding partners known to be selective for only one ATG8 protein [18] were analyzed for the presence and sequence of an LC3-interacting region (LIR motif). Canonical LIR motifs exhibit a small and rather loosely defined core sequence of four AAs: Y/F/W–X–X–V/I/L (X = any amino acid) (Figure 11C) [19]. Given the possible variability of positions 1 and 4, we hypothesized that the LIR motifs of the autophagic receptors recognized by different members of the Atg8 family might also differ at these positions. The results (Figure 11C) indicate that the amino acid sequences of the investigated LIR motifs were similar for all six mammalian ATG8 proteins. However, a certain degree of deviation was noted for LC3C-binding LIR motifs in terms of a less frequent usage of W in position 1 and a more frequent usage of hydrophobic or cationic AAs in position 3 (Figure 11C). This may be interpreted as a sign that LC3C is targeted by a partially different subset of autophagic receptors or interactors than LC3A and LC3B, supporting the idea of a sub- or neo-functionalization of LC3C during evolution.

## 4. Discussion

Autophagy is a key degradative pathway for maintaining cellular homeostasis [4,5]. Although autophagy has historically been described as a non-selective process, more recent evidence demonstrates that selective and thus cargo-specific forms of autophagy exist [2]. The microtubule-associated protein 1A/1B light chain 3B (LC3B) is arguably the most widely used marker of autophagic activity and autophagic flux, as it exhibits a posttranslationally modified, lipidated from that can be related to ongoing phagophore formation [2,6]. In humans, there are three paralogues of the LC3 gene family: LC3A, LC3B, and LC3C [7]. Despite the established importance of LC3B for the autophagic cascade, only a small number of studies have explicitly focused on LC3A and LC3C in terms of their cellular localization and regulation [26]. LC3C has been proposed to link the secretory and the autophagic pathway [27]. It has been further implicated in xenophagy and the specific targeting of mitochondria for mitophagy [28,29]. LC3A also seems to be involved in mitophagy since both proteins, LC3A and LC3C, have been related to Parkin-independent mitophagy pathways [30]. While LC3C has been described to target mitochondria through the outer mitochondrial membrane (OMM) protein metaxin 1, LC3A acts through binding to another OMM protein, FK506-binding protein 8 [31]. Autophagosomal cargo profiling has confirmed that upon BafA1 treatment, LC3A-, LC3B-, and LC3C-containing autophagosomes showed specificity for their cargo [29]. Besides the LC3 subfamily, three other members of the ATG8 family called GABARAPs have been described to play a role in different forms of autophagy that may occur independently of the LC3s [11,32,33]. 

In the present study, we analyzed LC3 lipidation as a marker for autophagic flux in primary human cells (diploid fibroblasts) in the presence of Ex527 and TSA, two deacetylase inhibitors known to act selectively on SIRT1 and non-sirtuin HDACs, respectively. These deacetylase inhibitors have been reported to show clear effects on LC3′s translocation from the nucleus into the cytosol [10], an effect that was recapitulated in this study (Figure 3, Figure 4 and Figure 7). With the exception of GABARAP-L2, two distinct bands could be resolved and quantified by Western blotting for each ATG8 protein. In analyzing the BafA1-induced difference in lipidation of the remaining five proteins as described [6], it was found that “LC3B-mediated autophagic flux” was severely compromised, as expected from LC3B’s intracellular distribution (Figure 1A,B). The same phenomenon was measured for GABARAP and GABARAP-L1. In contrast, “LC3A-mediated autophagic flux” was unaffected by both treatments trapping LC3B in the nucleus, while “LC3C-mediated autophagic flux” actually increased significantly in cells treated with Ex527 (Figure 1B). These changes were not brought about by a selective loss of the unlipidated proteins, but rather in the presence of stable (LC3A) or increased (LC3C) levels of the lipidated proteins (Figure 1). Based on these results, we conclude that the physiological regulatory mechanisms for LC3A and LC3C differ substantially from LC3B regulation, and that this differential response to acetylation might potentially serve as a buffering (LC3A) or compensatory (LC3C) mechanism to survive (local) LC3B insufficiency. The latter hypothesis was explored in detail by quantitative microscopic analyses summarized in the following. 

In immunostaining untreated primary human cells, we found that LC3B was mainly restricted to the nucleus, whereas LC3A (and p62) was primarily localized to the cytoplasm, while LC3C was stained in both compartments (Supplementary Figure S1). Upon BafA1 treatment, the majority of LC3B appeared in the cytoplasm to co-localize with p62, which was visible as vesicular structures and likely demarcated autophagosomes. At the same time, p62 was also co-localized with LC3A, while LC3C was found to be mainly migrating into the opposite direction into the nucleus; it was thus poorly co-localized with p62 (Figure 3 and Figure 4). Hence, both LC3A and LC3B would be appropriately distributed to engage in cytosolic autophagy under current basal conditions (i.e., with BafA1 treatment), whereas such a role for LC3C under these conditions appears unlikely. Still, provided that LC3B might also have a relevant function in the nucleus [10,24], LC3C would be suitable to replace it there. 

To test the idea a potential autophagic involvement LC3 proteins beyond LC3B, this protein was trapped inside the nucleus by preventing its nuclear export pharmacologically [10]. The according treatment with a selective SIRT1 inhibitor, Ex527, indeed led to a far-reaching loss of LC3B from the cytosol (Figure 3, Figure 4 and Figure 7) and a proportional loss of co-localization with p62, which would functionally involve autophagic insufficiency. In contrast, LC3A’s distribution was only moderately affected, and its almost complete co-localization with p62 was unchanged. LC3C, in turn, relocated actively into the cytosol and was proportionally more co-localized with p62. As total protein levels of all three LC3 proteins were either unchanged or increased in BafA1- and Ex527-treated cells (Figure 1), the described protein redistributions cannot be interpreted to have arisen from selective protein loss. For example, the 2.5-fold increased cytosolic fraction of LC3C in Figure 4D (and its proportional increase in p62 co-localization in Figure 4E) must have been accompanied by an absolute increase of LC3C protein in the cytosol since total protein levels of LC3C in parallel experiments analyzed by Western blotting were unchanged (Figure 1D). Interestingly, we observed an overall very similar pattern of redistributions with TSA, a non-sirtuin HDAC deacetylase inhibitor. As per quantitative image analysis and subcellular fractionation, LC3B was trapped in the nuclear regions as pronouncedly as with the selective SIRT1 inhibitor, Ex527 (Figure 3D, Figure 4D and Figure 7), even if the trapping may have been brought about by a different mechanism (suppressed dispersion in the cytoplasm through HDAC6 inhibition [22]). Nevertheless, very similar responses by LC3A and LC3C were induced, supporting the conclusion that these responses were secondary to functional LC3B insufficiency. Additional validation of our conclusions from these pharmacological experiments comes for the analysis of sirtuin 1 knock-down experiments, which again trapped mostly LC3B in the nucleus and increased the cytosolic association of only LC3A and LC3C with p62 (Figure 5). Moreover, the robustness and the LC3B-specificity of the acetylation regulatory mechanism were recapitulated under conditions of nutritional starvation (Figure 6). Finally, the distributional patterns of all LC3s and their redistributions in response to deacetylase inhibitor treatment were also verified in biochemical experiments, i.e., subcellular fractionation followed by Western blotting (Figure 7). 

In a third approach, we also investigated the differential response behavior of the three LC3 proteins in response to the genetic suppression of each of them by transfection with specific siRNAs (Figure 8, Figure 9 and Figure 10). Despite some technical interference (all proteins were less restricted to the nucleus at baseline even after scrambled RNA treatment), the fractional cytosolic association with p62 could be reliably quantified. The experiments confirmed the idea of a reciprocal and thus compensatory behavior of LC3B and LC3C under the assumption [10,24] that LC3B would have an essential function not only in the cytosol, but also in the nucleus. 

A sequence and binding partner comparison of the six human ATG8 proteins lends further plausibility to this idea. These proteins were confirmed to be highly conserved including their ATG7 binding sites and their common lipidation site, which may point towards a sub-functionalization rather than a neo-functionalization, which would usually involve addition of entire new functional domains (e.g., continuous sequence stretches at the termini or in the middle of one of the family members). This was generally not the case, but with the exception of short sequence stretches at both termini of LC3C (Figure 11B). In parallel with its behavior in cell culture, LC3C also stood out to some extent regarding its inferred interaction partners (Figure 11C), since the analyzed, putative LIR domains of its exclusive binding partners were distinguishable by their different occupation at AA positions 1 and 3. These slight, yet noticeable differences are well compatible with a functional differentiation of LC3C, albeit within the same paradigm, autophagy. Interestingly, many unique interaction partners of human ATG8s do not take part in autophagic processes, while many interaction partners that can bind to several different ATG8s do so. This could point to two things: first, the interaction with partners unrelated to autophagy may primarily drive their evolution. Second, ATG8 proteins are more likely to cross-compensate for the loss of another ATG8 with regard to autophagic processes rather than non-autophagic processes. 

In conclusion, the present study reveals a differential cellular localization and distributional regulation of LC3A and LC3C as compared to LC3B. LC3A and LC3C might have important autophagy-related functions due to their buffering (LC3A) and compensating (LC3C) ability of a lack of LC3B function. In general, LC3A is equidistributed between nucleus and cytosol, does not tend to travel much, but can associate more heavily with (cytosolic) p62 upon demand. LC3C, in turn, is highly mobile, and it always tends to migrate where LC3B has been decapacitated or removed, be it the cytosol or the nucleus. This behavior is indicative of a functional replacement. A significant decrease in the activity of sirtuins and HDACs in age-related diseases is widely recognized [34]. Hence, it may be a specific physiological task of LC3C to compensate for an LC3B loss-of-function in the cytosol due to age-associated sirtuin deficiency.

## Supplementary Materials

The following are available online at https://www.mdpi.com/2073-4409/9/10/2315/s1, Figure S1: Cellular localization of LC3A, LC3B, LC3C, and p62 proteins upon Ex527 or TSA treatment, Figure S2: Cellular distribution of p62 upon Ex527 or TSA treatment with or without treatment with BafA1, Figure S3: Acetylation status of LC3A, LC3B and LC3C upon Ex527 or TSA treatment, Figure S4: Analysis of the cellular localization of LC3A, LC3B and LC3C in response to siRNA-mediated Lamp2 knock-down under different conditions.
