# Supplementary material for: Novel Insights into the Cellular Localization and Regulation of the Autophagosomal Proteins LC3A, LC3B and LC3C
Source: Cells. 2020 Oct 18;9(10):2315. doi: 10.3390/cells9102315 (PMC7603224; doi:10.3390/cells9102315)
Supplement: Supplementary file 1 [file cells-09-02315-s001.pdf]

Supplementary Material

Novel Insights into the Cellular Localization and Regulation of the Autophagosomal Proteins LC3A, LC3B and LC3C

Marius W. Baeken, Katja Weckmann, Philip Diefenthäler, Jan Schulte, Kamran Yusifli, Bernd Moosmann, Christian Behl and Parvana Hajieva

Table of Contents

Supplementary Figure S1 page 2
Supplementary Figure S2 page 3
Supplementary Figure S3 page 4
Supplementary Figure S4 page 5

Supplementary Figure S1

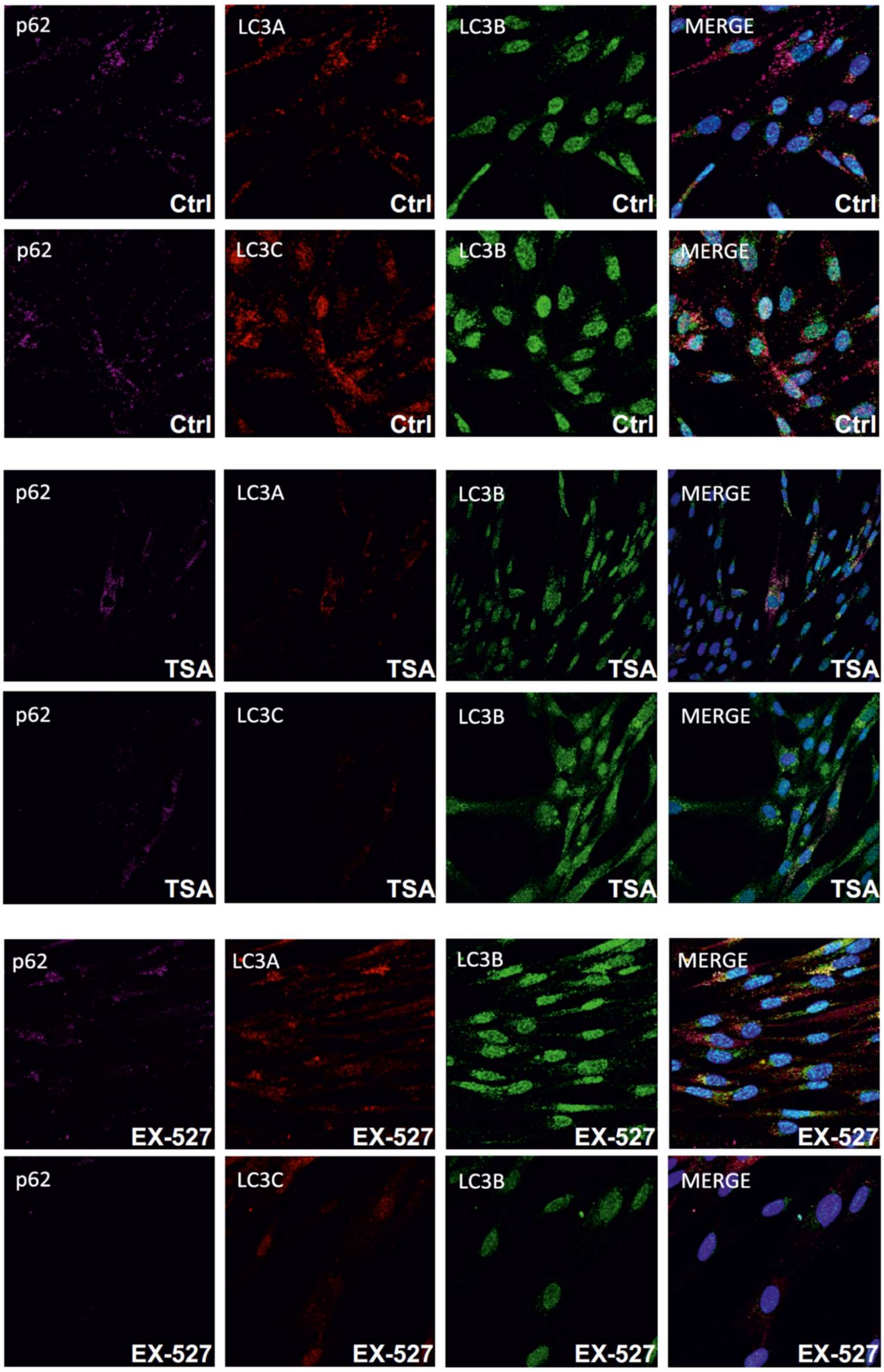

Supplementary Fig. S1. Cellular localization of LC3A, LC3B, LC3C, and p62 proteins upon Ex527 or TSA treatment. These cells had not been treated with BafA1.

Supplementary Figure S2

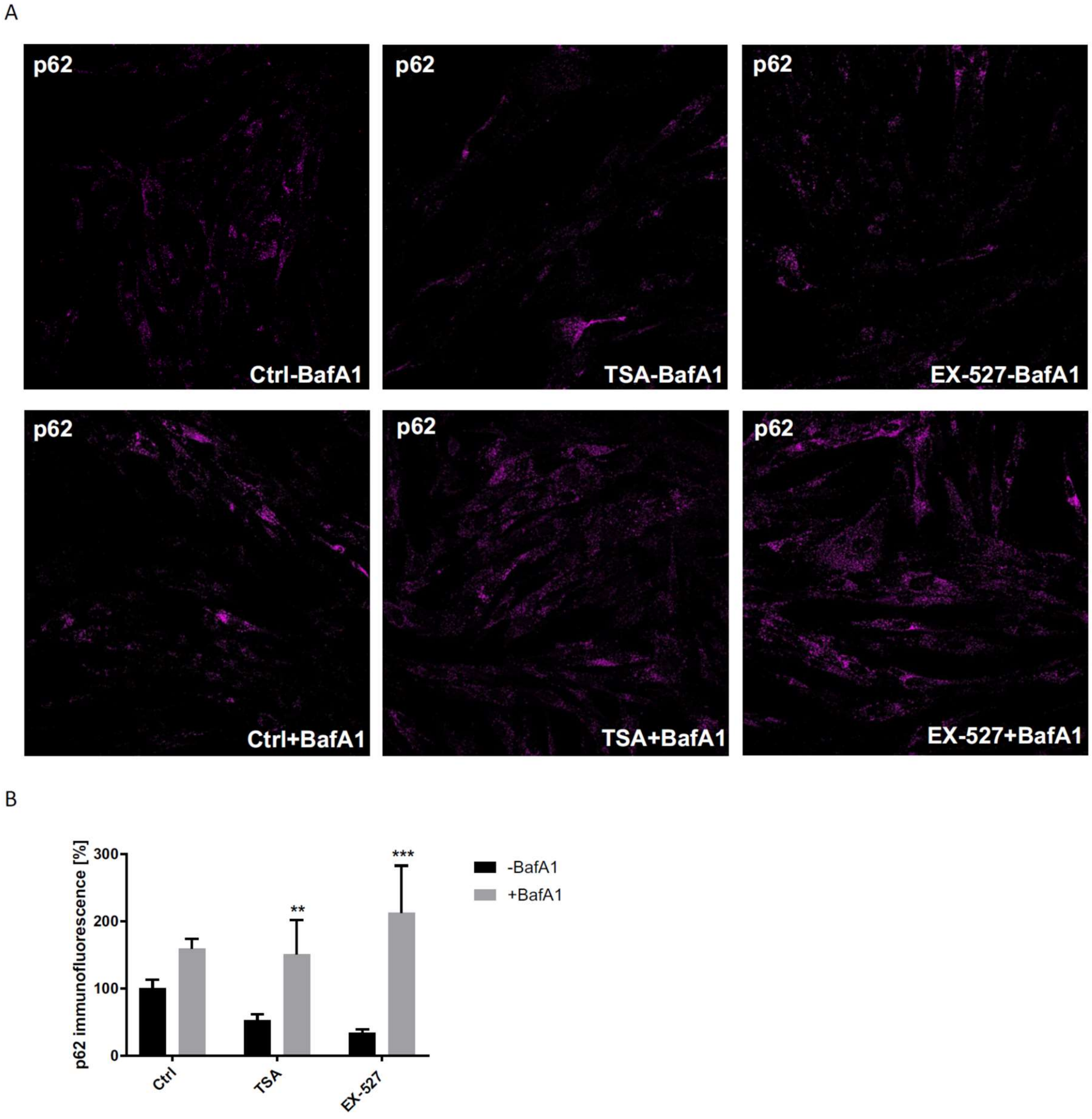

Supplementary Fig. S2. Cellular distribution of p62 upon Ex527 or TSA treatment with or without treatment with BafA1. (**A**) Representative pictures of p62-stained cells treated as indicated. (**B**) Quantification of total p62 staining intensity normalized on the number of cells per picture. Data in (**B**) were derived from three images per dish of three independent cell cultures (n = 3), in which 50-100 cells per image were analyzed. Significant changes (by one-way ANOVA) versus BafA1-untreated cells: **$p \leq 0.01$; ***$p \leq 0.001$.

Supplementary Figure S3

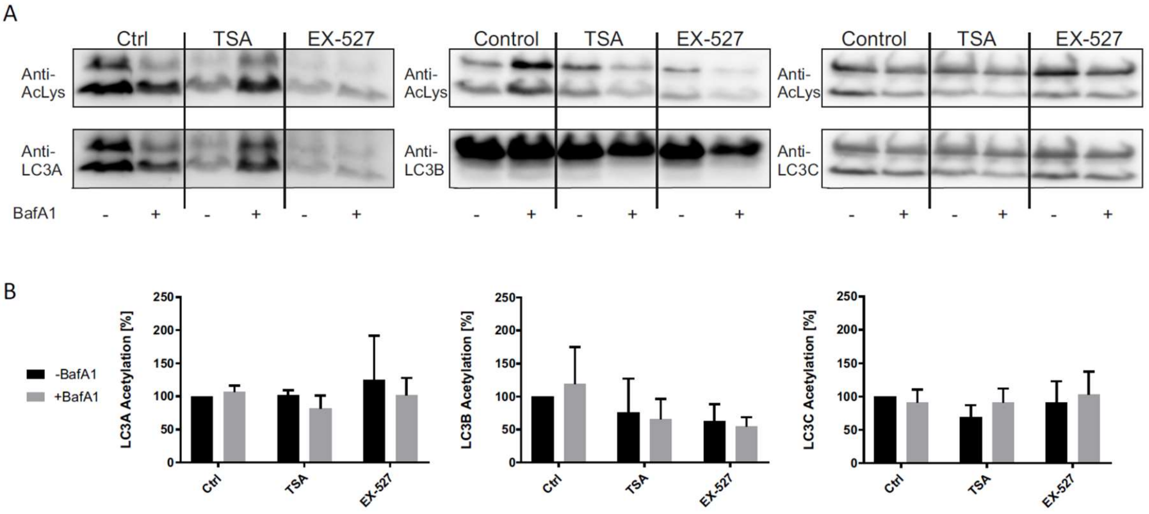

Supplementary Fig. S3. Acetylation status of LC3A, LC3B and LC3C upon Ex527 or TSA treatment. (**A**) Primary human fibroblasts were treated as indicated, and cell lysates were immunoprecipitated with antibodies against LC3A, LC3B and LC3C. The precipitates were investigated by Western blot with an antibody specific for acetylated lysine. (**B**) Quantification of LC3 acetylation as determined from n = 3 experiments.

Supplementary Figure S4

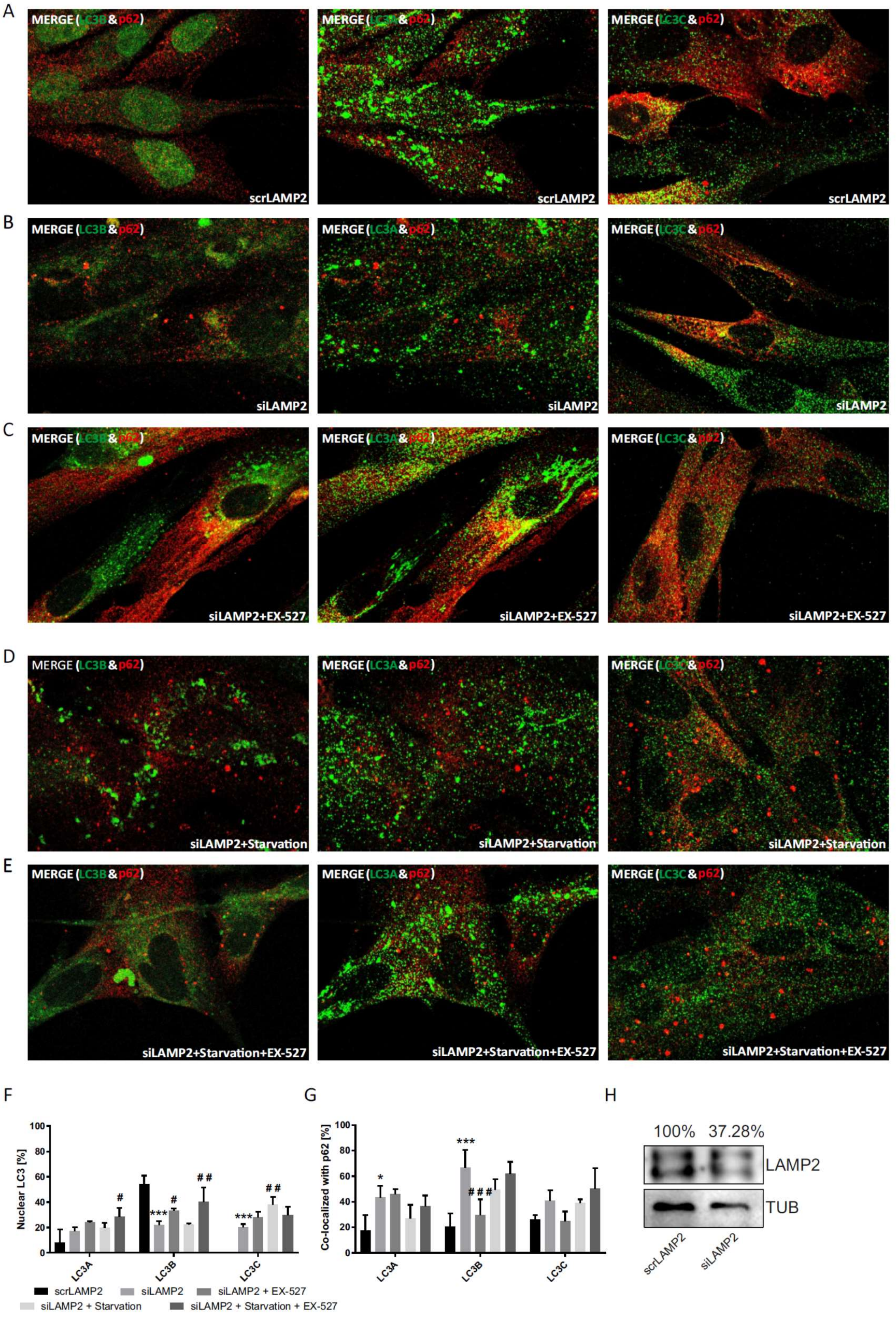

Supplementary Fig. S4. Analysis of the cellular localization of LC3A, LC3B and LC3C in response to siRNA-mediated Lamp2 knock-down under different conditions. Primary human fibroblasts were transfected with scrLamp2-RNA or siLamp2-RNA and treated with Ex527 or were starved as indicated. Four hours prior to immunocytochemistry, the cells were treated with BafA1. Subsequent analyses were performed by confocal fluorescence microscopy. (**A**-**E**) Representative images of cells transfected and treated as indicated. (**F**) Quantification of the relative nuclear fraction of LC3A, LC3B and LC3C. (**G**) Relative co-localization of the LC3s with p62 as quantified by image analysis. Significant changes (by two-way ANOVA) versus scrLamp2-RNA: $^{*}p \leq 0.05$; $^{***}p \leq 0.001$; significant changes versus siLamp2-RNA: $^{\#}p \leq 0.05$; $^{\#\#}p \leq 0.01$; $^{\#\#\#}p \leq 0.001$. Data in (**F**) and (**G**) were derived from three photographed, independent experiments (n = 3), in which 12-45 variably damaged survivor cells per image were analyzed. (**H**) Lamp2 protein expression in cells transfected with scrLamp2-RNA or siLamp2-RNA. Tubulin was used as loading control.
